# Value of copeptin and the S-100b protein assay in ruling out the diagnosis of stroke-induced dizziness pattern in emergency departments

**DOI:** 10.1186/s13049-019-0651-1

**Published:** 2019-08-06

**Authors:** N. Deboevere, N. Marjanovic, M. Sierecki, M. Marchetti, M. Dubocage, E. Magimel, O. Mimoz, J. Guenezan

**Affiliations:** 1 Service d’Acceuil des Urgences, SAMU 86, Centre Hospitalier Universitaire de Poitiers, 2 rue de la Milétrie, 86021 Poitiers, France; 2 INSERM U1070, Université de Poitiers, Poitiers, France

**Keywords:** Dizziness, stroke, copeptin, PS100b, Biomarker

## Abstract

**Background:**

Dizziness is a frequent reason for visiting emergency departments (EDs). Differentiating stroke from other causes is challenging for physicians. The role of biomarkers has been poorly assessed. We evaluated whether copeptin and S100b protein (PS100b) assessment, alone or in combination, could rule out stroke in patients visiting EDs for dizziness.

**Methods:**

We included patients 18 years of age or older, visiting the adult ED of a French university hospital for a new episode of dizziness evolving for less than 72 h. All patients underwent standardized physical examination (HINT [Head Impulse test, Nystagmus, test of skew deviation] maneuvers), copeptin and S-100b protein (PS100) measurement and injected brain imaging. Stroke diagnosis involved diffusion-weighted magnetic resonance imaging or, if not available, neurological examination and contrast brain CT scan compatible with the diagnosis.

**Results:**

Of the 135 patients participating in the study, 13 (10%) had stroke. The sensitivity, specificity and positive and negative predictive values of copeptin/PS100 combination were 100% (95%CI, 77–100%), 48% (40–57%), 14% (11–27%) and 100% (94–100%), respectively. Values for copeptin alone were 77% (CI95% 0.50–0.91), 50% (CI95% 0.49–0.58), 14% (CI95% 0.08–0.24), 93% (CI95% 0.87–0.98), and for PS100 alone were 54% (CI95% 0.29–0.77), 97% (CI95% 0.92–0.99), 64% (CI95% 0.35–0.84), 95% (CI95% 0.90–0.98).

**Conclusions:**

Absence of copeptin and PS100 elevation seems to ruling out the diagnosis of stroke in patients visiting the ED for a new episode of dizziness. These results need to be confirmed in a large-scale study.

## Introduction

Dizziness is a common reason for visiting emergency departments (EDs) worldwide [1, 2]. One of the main difficulties for the emergency physician is to distinguish stroke, which may require urgent neurovascular management, from peripheral etiologies of dizziness [1–5], whose treatment is mainly symptomatic. This difficulty is due to the subjective description of the dizziness by the patient and a lack of homogenized clinical examination by physicians. The three HINTS (Head Impulse Test, Nystagmus and Test of Skew Deviation) maneuvers have been proposed to clinically distinguish stroke from other etiologies, but the literature supporting these recommendations is limited [1, 6–13].

In this context, biomarkers could be useful. Copeptin is an endogenous stress marker, secreted by the pituitary gland, having good negative predictive value, particularly in the acute phase of myocardial infarction [14]. S-100b protein (PS100) is a marker of cerebral injury of vascular or traumatic origin [15–17]. Mainly secreted by astrocytes of the central nervous system, PS100 has good negative predictive value to rule out brain injury in mild head trauma [18]. Both biomarkers have been independently linked to severity of stroke [14, 19–24], and their concentrations increase in proportion to the National Institute of Health Stroke Score (NIHSS) value. Because of their different blood appearance kinetics [14–18, 25], early for copeptin and delayed for PS100, their negativity in the acute phase of dizziness has the potential to rule out stroke as its etiology.

We evaluated whether assessment of copeptin and PS100 protein, alone or in combination, in patients visiting an ED for newly developed dizziness could ruling out stroke as its cause.

## Methods

### Design of the study

We conducted a prospective, observational, monocenter study from May 1, 2016 to January 31, 2018 in the adult ED of the University Hospital of Poitiers after submitting the protocol to our local ethics committee (reference 2016–27).

### Patients

We included patients 18 years of age or older, visiting the ED for a new episode of dizziness evolving less than 72 h and having given written consent to participate in the study.

Non-inclusion criteria were head trauma during the last 72 h, capillary blood glucose level lower than 0.60 g/L on admission, toxic substance ingestion within 72 h of admission, chest pain or pathological electrocardiogram (heart rhythm disorders discovered in the ED), high-grade cardiac conduction disorders, Brugada and Wolff Parkinson White, unknown bradycardia with heart rate < 40/min, acute coronary syndrome).

Patients without brain imaging (diffusion-weighted magnetic resonance imaging (MRI) or contrast CT scan compatible with the diagnosis) were excluded.

### Procedures

We developed a protocol on the management of dizziness in an ED based on published scientific knowledge. Reminders on its application were regularly sent to residents and physicians working in the ED. This protocol included:

- Standardized and validated physical examination of dizziness (HINTS maneuvers) [6, 8, 13],
- Search for cerebellar ataxia,
- Search for disharmonious vestibular syndrome,
- Immediate call to a specialist (Neurologist or ENT specialist) when requested by attending emergency physician,
- Blood determination of PS100 and copeptin,
- Brain imaging: MRI alone, CT alone or both. Depending on clinical presentation, the imaging tests were performed during ED stay, during hospitalization or externally. Presence or absence of stroke was established on diffusion-weighted brain MRI [26]. In case of normal contrast CT alone, a specialized opinion should exclude the need for diffusion-weighted MRI according to clinical presentation.

### Data collection

Age, sex and time between dizziness onset and ED visit were collected using Resurgences® software (Intuitive, Berger-Levrault, Boulogne - Billancourt, France).

PS100 concentrations were measured on serum samples by electro-chemiluminescence assay (Roche, Mannheim, Germany). Copeptin concentrations were measured on serum samples by the Kryptor method (Thermo Scientific, Hennigsdorf, Germany).

The positivity threshold for copeptin was set, in accordance with the laboratory standard, at strictly above 10 pmol/L and that of PS100 was set at strictly above 0.105 μmol/L.

### Statistical analyses

All data were anonymized and analyzed with Excel® software (Microsoft corporation, Redmond, WA, USA). Quantitative variables were described by their median and interquartile and compared using Mann Whitney U test. Qualitative variables were described by their gross number and percentage and compared by a Chi-2 test. A value of *p* < 0.05 was considered significant. Sensitivity, specificity, and positive and negative predictive values predicting stroke as the cause of dizziness were calculated for each biomarker alone or in combination.

As this is an exploratory study, we included as many consecutive patients as possible during the inclusion period.

## Results

**Fig. 1:**
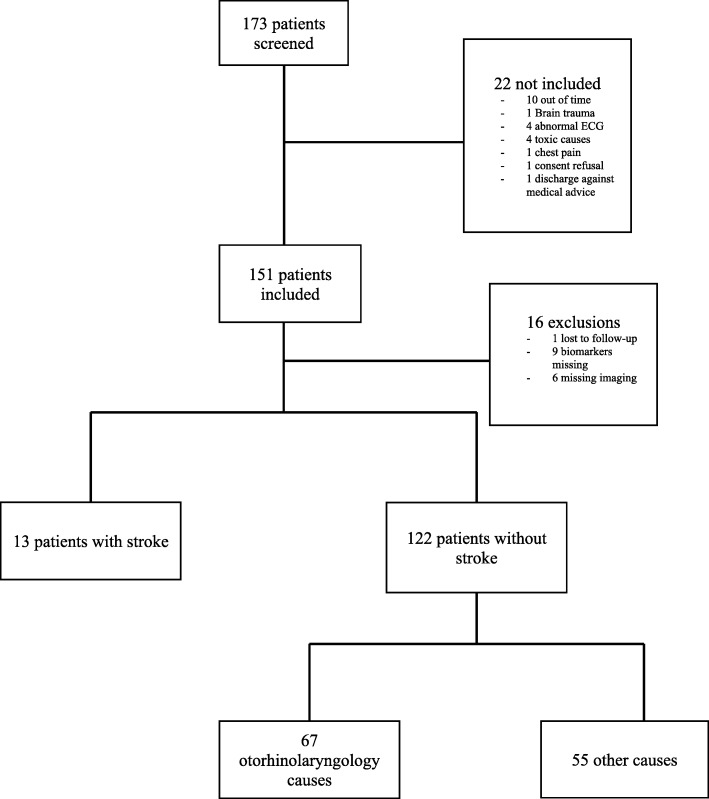
Study flowchart

One hundred and fifty-one patients were included, of whom 16 were secondarily excluded (lost to follow-up *n* = 1, missing biomarkers *n* = 9, missing MRI *n* = 6) (Fig. 1). A majority of the remaining 135 patients were women (*n* = 79, 59%) with an average age of 62 years. Specialized advice on the origin of dizziness was sought from ENT and/or neurologist in 102 (76%) of cases. Patients received brain diffusion-weighted MRI alone in 74 cases, contrast brain CT alone in 11 cases and a combination of MRI and CT in 50 cases.

In 122 patients (90%), vertigo was not related to stroke. Exclusion of this diagnosis was made on normal diffusion-weighted MRI alone or in combination with normal contrast CT in 95% of cases. The characteristics of patients with and without stroke are summarized in Table 1. Those with stroke causing dizziness were older. Conversely, there was no difference in gender and time between dizziness onset and clinical examination between the 2 groups.

**Table 1:** Patient characteristics, biomarker values and brain imaging type in patients with or without stroke as the main cause of a new episode of dizziness

	Stroke (*n* = 13)	No stroke (*n* = 122)	*P* value
Age (yrs)	70 [62–78]	60 [42–71]	0.023
Male sex	6 (46)	51 (42)	0.76
Delay between dizziness onset and ED visit (h)	12 [8.5–30]	12 [6–24]	0.395
Clinical data			
Positive HINT maneuvers	5 (38.5)	29 (24)	0.246
Positive nystagmus test	5 (38.5)	28 (3)	0.216
Positive HIT test	1 (7)	10 (8)	1
Positive skew test	1 (7)	1 (1)	0.184
Biomarker data			
Copeptin value (μmol/L)	70 [13.5–141]	10.5 [4–31]	0.065
Copeptin >normal value	10 (76)	61 (50)	0.002
S100 protein value (pmol/L)	0.11 [0.04–0.65]	0.05 [0.04–0.06]	< 0.0001
S100 protein >normal value	7 (54)	4 (3)	0.002
Brain imaging			
Tomodensitometry	5 (38)	6 (5)	0.001
Magnetic resonance imaging	3 (23)	71 (58)	0.02
Both	5 (38)	45 (37)	0.91

Results are expressed as median [Interquartile range] or number (percentage)

PS100 and copeptin concentrations above normal values were more frequent in patients having stroke (Table 1) than in those without stroke.

Table 2 shows the performance of copeptin and PS100, alone or in combination, to diagnose or rule out stroke as the cause of dizziness.

**Table 2:** Diagnostic values of copeptin and S100 protein, alone or in combination, to ruling out stroke as the main cause of a new episode of dizziness

	Sensitivity	Specificity	Positive predictive value	Negative predictive value
Copeptin	0.77 [0.50–0.91]	0.50 [0.49–0.58]	0.14 [0.08–0.24]	0.93 [0.87–0.98]
S100 protein	0.54 [0.29–0.77]	0.97 [0.92–0.99]	0.64 [0.35–0.84]	0.95 [0.90–0.98]
Both	1 [0.77–1]	0.48 [0.40–0.57]	0.14 [0.11–0.27]	1 [0.94–1]

Values in brackets are 95% confidence interval

## Discussion

In this study, absence of both copeptin and PS100 elevation effectively excluded stroke as the main cause of dizziness in patients visiting EDs for a new episode.

Dizziness is one of the most frequent symptoms occasioning ED visits. One goal for emergency physicians is to eliminate a neurovascular etiology that may require specific management as soon as possible. Typical clinic presentations are quickly referred to a neurologist, but atypical forms pose difficult diagnostic orientations, and may be responsible for treatment delay and worsened outcome. Biomarkers seem to provide diagnostic assistance in situations of uncertainty or difficulty in accessing specific imaging. However, delays in obtaining biomarker results do not currently justify their routine use in patients with highly probable diagnosis of stroke. Brain imaging is preferable, particularly in settings compatible with thrombolysis use. Our results are consistent with those previously reported by Purrucker and colleagues performed in similar patients [27]; in their study, PS100 concentrations were significantly higher in patients with stroke compared to those without (0.069 ng/ml versus 0.047 ng/ml, *p* < 0.001), with 94% sensitivity and 32% specificity for stroke diagnosis in cases of PS100 elevation.

The interest in combining the two biomarkers is explained by their different kinetic profiles. Copeptin rises in the first hours following endogenous stress and quickly decreases below normal values [19, 26, 28, 29]. Conversely, PS100 increase is delayed after stroke, until brain necrosis occurs, but lasts longer [25] .

Our study has several limitations. First, our population c**a**me from a monocentric cohort and the number of patients included (*n* = 135) was limited, with only 13 stroke episodes. Second, 11 (8%) included patients did not receive reference brain imaging (MRI). However, the risk of misdiagnosis is probably low since these 11 patients received contrast brain CT that was either abnormal (*n* = 5), thereby confirming the diagnosis of stroke, or normal (*n* = 6) but, following expert opinion, ruling out stroke.

To the best of our knowledge, our study is the first to examine the value of copeptin and PS100 used in combination as means of ruling out central origin of dizziness in patients visiting EDs for a new episode. Stroke was mainly identified on reference brain imaging. The use of biomarkers is probably not useful in situations where the diagnostic suspicion of stroke is high, especially in the early hours, when imaging diagnoses and guides therapeutic management. Conversely, they could be recommended in situations of uncertainty, where the emergency physician is often alone, with limited access to reference imaging. More studies with larger cohorts are required to confirm our results, and to better define the place of these biomarkers in the diagnostic strategy.

## Conclusions

Absence of copeptin and PS100 elevation seems to ruling out the diagnosis of stroke in patients visiting the ED for a new episode of dizziness. These results need to be confirmed in a large-scale study.

## Data Availability

All data generated or analysed during this study are included in this published article [and its supplementary information files].

## Abbreviations

CT: Computed tomography
EDs: Emergency departments
HINT: Head Impulse test, Nystagmus, test of skew deviation
MRI: Magnetic resonance imaging
NIHSS: National Institute of Health Stroke Score
PS100b: S100b Protein
